# Protective HLA-B57: T cell and natural killer cell recognition in HIV infection

**DOI:** 10.1042/BST20220244

**Published:** 2022-09-16

**Authors:** Christian A. Lobos, Jonathan Downing, Lloyd J. D'Orsogna, Demetra S.M. Chatzileontiadou, Stephanie Gras

**Affiliations:** 1 Department of Biochemistry and Chemistry, La Trobe Institute for Molecular Science, La Trobe University, Bundoora, VIC 3083, Australia; 2 Department of Biochemistry and Molecular Biology, Monash University, Clayton, VIC 3800, Australia; 3 School of Biomedical Sciences, University of Western Australia, Nedlands, WA 6009, Australia; 4 Department of Clinical Immunology and PathWest, Fiona Stanley Hospital, Murdoch, WA 6150, Australia

**Keywords:** CD8^+^ T cell, HIV, HLA-B57, immune response, natural killer cell, peptide presentation

## Abstract

Understanding the basis of the immune determinants controlling disease outcome is critical to provide better care to patients and could be exploited for therapeutics and vaccine design. The discovery of the human immunodeficiency virus (HIV) virus as the causing agent of acquired immunodeficiency syndrome (AIDS) decades ago, led to a tremendous amount of research. Among the findings, it was discovered that some rare HIV^+^ individuals, called HIV controllers (HICs), had the ability to control the virus and keep a low viral load without the need of treatment. This ability allows HICs to delay or avoid progression to AIDS. HIV control is strongly associated with the expression of human leukocyte antigen (HLA) alleles in HICs. From the HIV protective HLAs described, HLA-B57 is the most frequent in HIC patients. HLA-B57 can present a large range of highly conserved Gag-derived HIV peptides to CD8^+^ T cells and natural killer (NK) cells, both the focus of this review. So far there are limited differences in the immune response strength, magnitude, or receptor repertoire towards HIV epitopes that could explain viral control in HICs. Interestingly, some studies revealed that during early infection the large breadth of the immune response towards HIV mutants in HLA-B57^+^ HIC patients, might in turn influence the disease outcome.

## Introduction

Human immunodeficiency virus (HIV) directly weakens the immune system [1] and currently ∼38 million people live with the virus worldwide (https://www.who.int/teams/global-hiv-hepatitis-and-stis-programmes/hiv/strategic-information/hiv-data-and-statistics). Antiretroviral therapy (ART) has dramatically improved the health of HIV-infected individuals; however, many side effects arise from prolonged ART use [2,3]. Furthermore, only three-quarters of all people living with HIV have access to ART, leaving ∼10 million people to live without, including ∼50% of HIV^+^ children (www.unaids.org/sites/default/files/media_asset/2022-global-aids-update_en.pdf). There is urgent need to develop new treatments and ideally an HIV vaccine. The major hurdle towards a cure or a vaccine against HIV is due to the genetic diversity of the virus that allows for evasion of immune surveillance [4,5] and post-infection latency creating a viral reservoir [6]. This ability to ‘hide’ leads to persistent infection that weakens the immune system and causes progression to acquired immunodeficiency syndrome or AIDS [7].

Rare individuals termed HIV controllers (HICs) or long-term nonprogressors (LTNP), can maintain low viral loads (<50 copies of HIV RNA/ml of plasma) and remain healthy in the absence of ART [8,9]. HICs only represent <0.5% of HIV^+^ individuals. It remains unknown how viral control is achieved. Therefore, understanding the basis of HIV control in HIC individuals could offer clues to provide better protection and potentially eliminate the virus by mimicking, or even boosting the immune response.

Interestingly, the strongest association with HIV control is the expression of ‘protective’ human leukocyte antigen (HLA) alleles [10], which is in turn linked to a functionally superior CD8^+^ T cell response. This is mediated by the T cell receptor (TCR) recognition of the HLA presenting HIV epitopes. Some protective HLAs against HIV are HLA-B57 [11,12], HLA-B27 [13], HLA-B81 [13], and HLA-B52 [14]. However, other HLA molecules have been linked with progression to AIDS and can be detrimental in progressor patients.

HLA-B57 is found at a global frequency of 1% [15] but >40–60% in HIC individuals [11]. Studies have discerned that HLA-B57-mediated HIV control is associated with strong CD8^+^ T cell responses early on in HIV infection [11,16], expansion of immunodominant T cell clones [11,17], and recognition of conserved HIV epitopes [17]. In addition to T cell-mediated response, HLA-B57 can also present HIV peptides to natural killer (NK) cells. There are links between viral control in HLA-B57^+^ individuals and the expression of specific NK cell receptor allomorphs [18]. However, it needs to be clarified that although the HLA-B*57 allele is highly associated with the HIC phenotype, it is not sufficient by itself to confer control of viremia [11].

In this review, we explore the characteristics of the HLA-B57 molecule and its association with HIV control based on the presentation of conserved Gag epitopes targeted by both CD8^+^ T cells and NK cells.

### Characteristics of the most HIV protective HLA allele: HLA-B57

HLA molecules are highly polymorphic, with >24 000 HLA class I alleles described to date [19,20]. HLA molecules consist of a heavy and a light chain (α and β2-microglobulin, respectively). The α-chain forms the peptide binding cleft that accommodates small peptides (usually 8–10 residues) [21,22]. The peptide binds to the HLA cleft via six pockets designated A through F [21] and is anchored by two primary residues, one at position 2 (P2) and one being the last residue (PΩ), that bind the B and F pocket, respectively [21].

**Figure 1.**
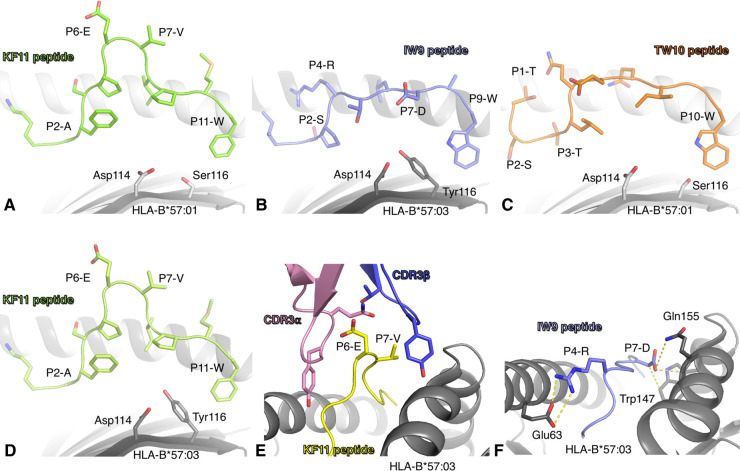
Structures of KF11 and IW9 presented by HLA-B57. (**A**) Structure of HLA-B*57:01 (pale grey cartoon) presenting the KF11 peptide (green sticks). (**B**) Structure of HLA-B*57:03 (grey cartoon) in complex with the IW9 peptide (blue sticks). (**C**) Structure of HLA-B*57:01 (pale grey cartoon) with the TW10 peptide (orange sticks). The shift towards the N-terminal of the TW10 peptide forces the P1-T outside the cleft. (**D**) Structure of the HLA-B*57:03 (grey cartoon) presenting the KF11 peptide (light green sticks). (**A–D**) The polymorphic residues of HLA-B57 (positions 114 and 116) are shown as sticks. (**E**) Structure of the AGA1 TCR (CDR3α in pink and CDR3β in blue) docking atop the HLA-B*57:03 (grey cartoon) presenting the KF11 peptide (yellow cartoon and sticks). (**F**) Side view of HLA-B*57:03 cleft (grey cartoon) showing the interactions (yellow dashed lines) between the HLA residues (grey sticks) and the IW9 peptide (blue cartoon and sticks).

The most frequently expressed HLA-B57 alleles are HLA-B*57:01 (1%), HLA-B*57:02 (0.1%), and HLA-B*57:03 (0.4%) [15] with some differences based on ethnicity (Table 1) [23]. Polymorphisms between the three HLA-B57 allomorphs are located at positions 114, 116, and 156 (Table 2). Residues 114 and 156 are in the C pocket. Polymorphic residue 114 can impact the peptide secondary anchor residue [21,24], while residue 116 could affect the binding of the primary anchor residue to the F pocket (Figure 1A) [21,24].

**Table 1.** Frequency of the main HLA-B57 allomorphs

	Africa (%)	Asia (%)	Europe (%)	North America (%)	Central and South America (%)
HLA-B*57:01	10	7	6	1.8	1.6
HLA-B*57:02	2	0.1	0.1	0.2	0.6
HLA-B*57:03	5	0.4	0.7	0.8	0.9

The data were obtained from the server allelefrequencies.net.

**Table 2.** Polymorphisms within the main HLA-B57 allomorphs

	HLA residue position		
	114	116	156
HLA-B*57:01	D	S	L
HLA-B*57:02	**N**	**N**	**R**
HLA-B*57:03	**Y**	**Y**	—

Polymorphic residues are shown in bold and matching residue is shown as dash.

HLA-B*57:01 and HLA-B*57:03 have been associated with HIV control in Caucasian and African patients, respectively [25–27]. Both allomorphs are associated with slower HIV-1 disease progression and present a broad range of conserved CD8^+^ T cells epitopes [28]. Although HLA-B*57:02 was identified in an African population [29], there is limited information beside the fact that polymorphisms change the peptide repertoire compared with the other two allomorphs [30]. The impact of polymorphisms on HIV control has been described, with HLA-B*57:01 being the most protective allomorph and HLA-B*57:03^+^/HIV^+^ patients exhibiting lower viral load than HLA-B*57:02^+^ patients [31].

### HLA-B57-restricted HIV-specific CD8^+^ T cell response

There is a total of 37 published HIV-derived epitopes that are presented by HLA-B*57:01 [32]. The majority of the studied HIV epitopes are derived from the Gag-Pol polyprotein due to the strong T cell response associated with them. The link between protection against AIDS progression and HLA-B57 was proposed to be due to the large number of Gag-derived epitopes that this HLA can present. Among the Gag epitopes, three are highly conserved and widely studied, namely IW9 (^138^ISPRTLNAW^146^), KF11 (^162^KAFSPEVIPMF^172^), and TW10 (^240^TSTLQEQIGW^249^) (Figure 1, Table 3) [33].

**Table 3.** Crystal structures available for the three conserved Gag-derived HLA-B57-restricted epitopes

Epitope name	Epitope sequence	HLA	TCR or KIR	PDB code
KF11				
KF11	^162^KAFSPEVIPMF^172^	HLA-B*57:01		2YPK [46]
KF11	^162^KAFSPEVIPMF^172^	HLA-B*57:03		2BVO [47]
KF11	^162^KAFSPEVIPMF^172^	HLA-B*57:03	AGA1 TCR	2YPL [46]
IW9				
IW9	^138^ISPRTLNAW^146^	HLA-B*57:03		2BVP [47]
TW10				
TW10	^240^TSTLQEQIGW^249^	HLA-B*57:01		5V5M [61]
TW10	^240^TSTLQEQIGW^249^	HLA-B*58:01		5V5L [61]
TW10	^240^TSTLQEQIGW^249^	HLA-B*57:01	KIR3DL1	5T6Z [62]
T242N (TW10)	^240^TS**N**LQEQIGW^249^	HLA-B*57:01	KIR3DL1	5T7O [62]

HIV-epitope mutation is highlighted in bold.

There is conflict in the literature. On the one hand, there are studies that show no differences in the CD8^+^ T cell responses, between HLA-B57^+^ HICs and progressors, specific to HLA-B*57:01-restricted epitope, or the restriction of virus replication [34]. On the other hand, other studies report differences in the frequency of polyfunctional CD8^+^ T cells [35], clonotype numbers or diversity for KF11- and IW9-specific T cells [36]. Despite this, some rare HIV mutants were observed only in HICs, suggesting a potential selective pressure by CD8^+^ T cells in HICs [37]. The consensus so far suggests that the CD8^+^ T cell response in HICs is less broad than in progressors, due to a high focus on HLA-B57-restricted epitopes [11,34]. The success of T cells in cancer immunotherapy [38] has led to a resurgence of interest to characterise and compare HIV-specific T cells in HICs and progressors, exploring new protective HLA alleles [39–41].

While the mechanism behind the critical role of T cells in HIV control is unclear, the current data suggest that there is more than one mechanism for viral control, such as NK cells discussed below.

### T cell recognition and HLA-B57 presentation of the ‘featured' KF11 epitope

The KF11 peptide was identified as a CD8^+^ T cell epitope in HLA-B*57:01^+^/HIV^+^ individuals (75% of HICs) and is immunodominant during chronic infection [17]. The T cell response towards KF11 epitope is stronger compared with other HIV epitopes in HLA-B57^+^ patients and is associated with lower viral load [42,43]. Despite HLA-B57 allomorphs being able to present the KF11 epitope, some differences were observed in the breadth and composition of the viral mutants, with rarer mutations found in HLA-B*57:01^+^ patients [44]. The increased diversity of KF11 variants identified in HLA-B*57:03^+^ patients was associated with higher viral load [45]. In HIC individuals T cells could cross-recognise KF11 variants [28], with some exceptions such as the A163G/S165N mutant, which is poorly recognised by T cells [46]. Interestingly, the A163G/S165N mutation is reversed in the absence of HLA-B*57:03 [46]. This shows that a strong T cell pressure can limit viral escape or trigger loss of viral fitness, and likely contribute to viral control in HLA-B57^+^ patients.

The T cell response differences observed between the HLA-B57 allomorphs were not due to conformational differences in the presentation of the KF11 epitope. The crystal structures of both HLA-B*57:01-KF11 [47] and HLA-B*57:03-KF11 [48] are similar (Figure 1A,D). Due to its length of 11 residues [21,22], the central part (P6–P7) of KF11 peptide protrudes outside the HLA binding cleft, likely being a potential target for TCR binding [22,49]. The TCR repertoire of KF11-specific T cells revealed a biased gene usage towards TRBV19 and TRBV7 [36,50]. While there was no significant difference in the TCR repertoire between HICs and progressors [36], HLA-B57 allomorph-specific differences were observed once again. A study showed that the KF11-specific TCR repertoire in HLA-B*57:03^+^/HIV^+^ patients was more diverse and less cross-reactive than in HLA-B*57:01^+^ patients [50]. This could be linked with the observation that a public TCR (shared between individuals) called AGA1 TCR (TRAV5/TRBV19) had a 5-fold higher affinity for HLA-B*57:01-KF11 compared with HLA-B*57:03-KF11 [47]. The AGA1 TCR was representative of the conserved CDR3α and β motifs identified in HLA-B*57:01^+^ HIC individuals, while absent from HLA-B*57:03^+^ donors [28,50,51].

The crystal structure of the AGA1 TCR in complex with HLA-B*57:03-KF11 shows a large contribution of the biased Vβ-chain [47], and the KF11 protruding central part is wrapped up by the CDR3 loops (Figure 1E). The TCR binding resulted into a push of the KF11 protruding part towards the HLA α1-helix and a cleft conformational change, which was associated with a long association rate [47]. While the AGA1 TCR structure was not solved in complex with the HLA-B*57:01-KF11, given the similarities of the HLA-B57-KF11 structures it is likely that the TCR binds in the same fashion to both allomorphs. However, the AGA1 TCR affinity differences towards the HLA-B57 allomorphs that were determined, were due to the thermodynamic interaction and the impact of polymorphic residues on the water-mediated interaction network [47]. It has been shown that water molecules in conjunction with polymorphic residues can contribute to the plasticity, orientation and flexibility of the bound peptide [52]. Therefore, minor variations of buried water molecule networks in association with polymorphic residues while allowing for the same peptide conformation observed in both allomorphs could lead to different peptide flexibility. This could in turn change the way T cells engage with the same peptide presented by a different allomorph and alter the resulting T cell repertoire, as shown for the KF11-specific TCR repertoire in HLA-B*57:01^+^ and HLA-B*57:03^+^ individuals [28,50,51].

Altogether, the T cell response to the KF11 epitope shows that even subtle differences within the protective HLA-B57 allomorphs can deeply influence the disease outcome.

### Features of T cell specific for the rather ‘flat' IW9 epitope

Similarly to the KF11 epitope, the IW9 (^138^ISPRTLNAW^146^) can stimulate CD8^+^ T cells in HIV^+^ patients that express HLA-B*57:01^+^, HLA-B*57:02^+^, HLA-B*57:03^+^, or HLA-B*58:01^+^ [36,42,53–55]. While IW9 can bind to HLA-B*58:01 molecule, like KF11, the T cell activation is not as strong as in HLA-B57^+^ individuals, and there are limited escape mutants detected [31]. IW9-specific T cells were detected in HICs and progressors, regardless of ART treatment, in contrast with other HIV epitopes such as QW9 for which specific T cells are only detected in HICs [36,54].

Two previous studies compared a few IW9-specific TCRs in HLA-B57^+^ HICs and progressors and could not distinguish between the two groups [36,54]. A biased usage of TRBV27 and TRBV7–8/7–9 gene families was observed (information available only for the β-chain). A recent study also described the lack of contrast in the TCR repertoire between HICs and progressors and observed the same biased gene usage towards TRBV7–9 as well as TRBV4–1 with some conserved CDR3β motifs [42].

The structure of IW9 peptide was solved in complex with HLA-B*57:03 (Figure 1B) [48]. The IW9 epitope's conformation is in stark contrast with the one observed for the longer KF11 epitope. IW9 has a rather flat structure conformation, despite having residues with large side chains such as P4-Arg and P7-Asp. The P4-Arg side chain is facing down forming a salt bridge with the Glu63 of the HLA (Figure 1F). The P7-Asp side chain lays flat in between the peptide backbone and the HLA α1-helix (Figure 1F).

The molecular basis of recognition of the rather ‘flat' IW9 peptide by a TCR is unknown. Future research should explore the structural basis of the response to this epitope which it could in turn provide some explanation for the biases observed in the T cell repertoire [42]. This would provide insights into the molecular mechanisms that underpin the T cell biology which defines the immune response towards this epitope.

### TW10 is presented with an unusual conformation in HLA-B57 binding cleft

The T cell response to the TW10 epitope (^240^TSTLQEQIGW^249^) is shown to play an important role in viral-load control in early HIV infection that might influence the disease outcome [33,43,56]. TW10 has been shown to be frequently targeted by T cells, rapidly mutate, escape and revert, in line with the observation that TW10 is targeted early in acute HIV infection [44].

Despite the potentially significant role of the TW10 epitope in acute infection, T cell repertoire information for this epitope is limited [42,57,58] and does not allow for comparison between HICs and progressors. The strong TW10 T cell response in early infection, results in the rapid mutation to a common escape mutant at position 242 (T242N) [44,59]. The T242N mutation decreases the viral replication capacity and the viral fitness [60]. Another TW10 mutation, G248A, decreases the T cell response without decreasing the viral fitness [59] but rather increases the viral infectivity [61].

**Figure 2.**
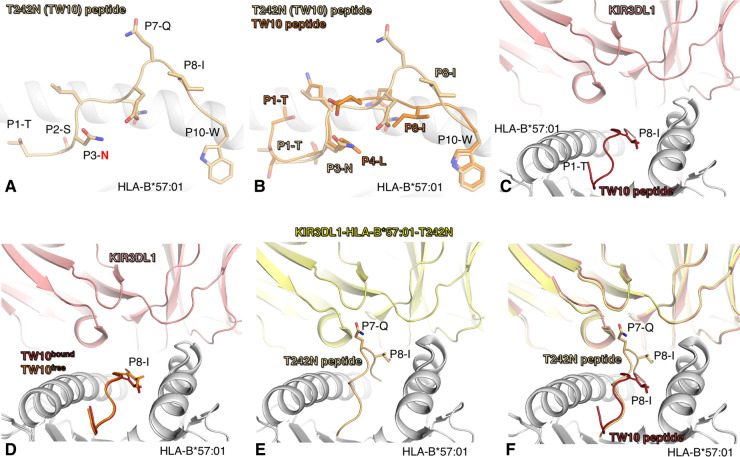
Structure of TW10 presented by HLA-B*5701 and recognised by KIR3DL1. (**A**) Structure of HLA-B*57:01 (pale grey cartoon) presenting the T242N mutant peptide of TW10 (pale orange stick and cartoon). The T242N at position 3 of the TW10 peptide is labelled in red on the panel. (**B**) Superposition of the HLA-B*57:01 (pale grey cartoon) structures in complex with the TW10 (orange) and T242N (pale orange) peptides. (**C**) Structure of the complex between the KIR3DL1 (pink cartoon) docking onto the HLA-B*57:01 (pale grey cartoon) presenting the TW10 peptide (red cartoon). (**D**) Superposition of the HLA-B*57:01 (pale grey cartoon) presenting the TW10 peptide free (orange cartoon) or the TW10 peptide bound to the KIR3DL1 (red cartoon). (**E**) Structure of the complex between the KIR3DL1 (pale yellow cartoon) docking onto the HLA-B*57:01 (pale grey cartoon) presenting the T242N peptide (pale orange cartoon). (**F**) Superposition of the KIR3DL1-HLA-B*57:01-TW10 structure with the KIR3DL1-HLA-B*57:01-T242N structure using the same colour scheme as per panel (**C**) and (**E**).

The structure of the HLA-B*57:01 presenting TW10 revealed an unusual conformation of the peptide (Figure 1C, Table 3). Instead of using the P2 residue as primary anchor [21], the TW10 binds to the HLA-B*57:01 cleft via the P3-Thr residue (instead of P2-Ser). This ‘shift' in the peptide pushes the P1-Thr residue outside the binding cleft pointing upwards, instead of binding to the A pocket of the HLA (Figure 1C) [62]. A similar shift of the P3-Thr is also observed when the TW10 peptide is presented by HLA-B*58:01 molecule [62]. Surprisingly, the T242N mutant adopts a canonical conformation (Figure 2A,B), whereby the P2-Ser is a primary anchor and binds into the B pocket [63]. As a result, there was large displacement of the same residue between the two peptides, with a distance of 3.9 Å between the P7-Gln Cα atoms and of 2.8 Å for the P8-Ile Cα atoms (Figure 2B). The structure of the T242N binding to HLA-B*57:01 is not free but in complex with a natural killer TCR (KIR3DL1) that might impact on the structure of the peptide (Table 3).

It remains to determine how T cells recognise TW10, with the structure of T242N peptide providing a potential basis for the lack of recognition by TW10-specific T cells due to the different conformation.

### Natural killer cell receptor recognition of HIV epitopes presented by the HLA-B57 molecule

Natural killer (NK) cells and their surface receptor killer immunoglobulin like receptor (KIR) [64], in the context of HIV infection, have been shown to mediate an anti-viral effect. The KIR receptors are divided into two groups: inhibitory or activating, depending on the cytoplasmic tail of the receptor, either long (L) or short (S) [65]. Many studies report specific KIR genes, in particular the presence of KIR3DL1 or KIR3DS1, to be associated with protection from HIV infection in patients expressing certain HLA-B molecules [18,66]. Functional evidence for NK cell-mediated inhibition of HIV-1 replication was demonstrated [67] and was also associated with distinct KIR and HLA allomorphs. NK cells expressing KIR3DS1 showed strong and cell contact-dependent inhibition of HIV-1 replication within cultured HIV-infected CD4^+^ T cells. The presence of KIR3DS1 was associated with increased NK cell activation and degranulation. In addition, HIV peptides were shown to enable the specific interaction of KIR3DS1 with HLA-B*57:01 [68]. Recently, a variant of the KIR3DL1 molecule was shown to be associated with HIV control and delayed disease progression in HLA-B57^+^ patients [18]. KIR3DL1 polymorphism (I47V) was the only significant modifier of the HLA-B57-mediated HIV protection identified by whole genome sequencing. This effect was specific to the HLA-B*57:01 and not observed for the HLA-B*57:03 allomorph. This suggested a direct interaction between KIR3DL1 and HLA-B57.

Recently, the crystal structures of KIR3DL1 in complex with both TW10 and T242N presented by HLA-B*57:01 were solved (Table 3) [63]. The interaction of the KIR3DL1 is mainly focussed on HLA-B*57:01-TW10 and located above the C-terminus of the HLA cleft and TW10 peptide (Figure 2C). Comparison of the HLA-B*57:01-TW10 structure with and without the KIR3DL1 revealed that the KIR binding pushes the P8-Ile of the peptide deeper within the HLA cleft (Figure 2D) [62,63]. This structural change occurs without close contact of the KIR3DL1 with the peptide (contact distance >4 Å). The KIR3DL1 structure in complex with the T242N mutant revealed, surprisingly, a similar docking mode to that with TW10 (Figure 2E,F). The KIR3DL1 binds to both peptides presented by HLA-B*57:01 with moderate to low affinity [63]. However, cells expressing KIR3DL1 only weakly bind to the HLA-B57-T242N tetramer showing that subtle differences can impact the immune cell recognition [63]. This lack or low interaction with HLA-B57-T242N tetramer was specific to the expressed KIR allotype, showing the importance of KIR polymorphisms in the control of HIV.

The ability of both KIR and T cells to recognise HIV epitopes bound by HLA-B57 could potentially reduce viral escape in HLA-B57^+^ patients.

## Conclusions

Despite the research interest in protective HLAs, such as HLA-B57, including their role in HIV control and the potential to advance new therapeutics, there are still many questions remaining. Surprisingly, even for conserved potent Gag-derived epitopes such as TW10, KF11, and IW9, there are gaps in knowledge regarding T cell function, cross-reactivity patterns, TCR repertoire diversity, affinity, and magnitude of the response. These parameters are critical to enable a clear comparison between HIC and progressor individuals and to understand if the differences occur early during infection as previously suggested [33,44]. Differences, between HICs and progressors, in T and NK cells’ cross-reactivity early during infection might lead in a decreased viral fitness in HIC individuals that would help the immune system to efficiently control HIV [58]. In other words, T cells and NK cells that recognise HLA-B57 presenting HIV epitopes in early infection might ‘buy time' for the immune system in HIC individuals.

There are some differences between HICs and progressors, but not significant yet to draw any conclusions. A recent study showed that across HIV epitopes presented by HLA-B57, progressors were selecting TCRs expressing the TRBV7-9 gene family more than HIC individuals [42]. Given the differences in viral load and disease outcome between HLA-B*57:01^+^ and HLA-B*57:03^+^ individuals, despite presenting peptides in similar fashion, there are differences that T cells can ‘detect' and provide an advantage to HICs. While the mechanisms are not fully clear, HLA-B57 molecules provide an advantage in HIV infection as well as in HCV infection [69] and therefore more research on HLA-B57 will be of interest. Some studies also suggest that there are some TCR-independent factors that modulate CD8^+^ T cell function such as epigenetic modifications that allow for effective function in chronic HIV infection [70]. Several studies involving HLA-B*57:01^+^ HIC individuals have shown that the host immune factors might drive the restriction of viral replication, while the mechanism remains unclear [11,34].

The research reviewed here clearly highlights the subtle differences in the epitope presentation of the HLA-B57 allomorphs which impact their recognition by T cell and NK cell receptors. In addition to the highly polymorphic nature of HLA molecules, KIR receptors on the surface of NK cells are also polymorphic, similar to TCRs, which adds some layer of complexity in the search for a common feature that could define the basis of the HLA-B57 protective nature. These factors combined seem to have, to date, prevented the development of interventions based on the protection offered by HLA-B57 carriage in some individuals. However, further dissection of the immune response in HIV-1 controllers may yield insights to allow for the successful modulation of the immune response to afford this protection to broader populations.

**Perspectives**

- Better understanding of the immune response to protective HLAs, such as HLA-B57, and their role in HIV control has the potential to advance new therapeutics.
- The data currently available on protective HLA alleles does not allow to conclude on the mechanism for viral-load control observed in HIC individuals.
- More work on exploring the basis of HIV control is worth pursuing as this could reveal some critical information to better fight HIV.

## Abbreviations

AIDS: acquired immunodeficiency syndrome
ART: antiretroviral therapy
HIV: human immunodeficiency virus
HLA: human leukocyte antigen
KIR: killer immunoglobulin like receptor
NK: natural killer
TCR: T cell receptor
